# Autophagy and apoptosis-related genes in chronic liver disease and hepatocellular carcinoma

**DOI:** 10.1186/1471-230X-12-118

**Published:** 2012-08-28

**Authors:** Andromachi Kotsafti, Fabio Farinati, Romilda Cardin, Umberto Cillo, Donato Nitti, Marina Bortolami

**Affiliations:** 1 Department of Surgery, Oncology and Gastroenterology Division of Gastroenterology, University of Padova Via Giustiniani 2, 35128, Padova, Italy

**Keywords:** Autophagy, B and C virus infection, Beclin 1, Hepatocellular carcinoma, Pro- and anti-apoptotic factors.

## Abstract

**Background:**

Dysregulation of autophagy is important in the pathogenesis of many diseases, including cancer. Several aspects of the biological role of autophagy are however still unclear and the relationship between apoptosis and autophagy, particularly in the liver has yet to be thoroughly explored. In this study we evaluated the expression of Beclin 1 (one of the main autophagocytic agents, which bridges autophagy, apoptosis and both differentiation), and both pro- (Bad, Bax) and anti-apoptotic (Bcl-2, Bcl-xL) factors in liver samples from patients with different stages of liver disease.

**Methods:**

The study concerned 93 patients from 49 cases of chronic hepatitis (CH) (30 HCV and 19 HBV-related), 13 of cirrhosis (CIRR) (10 HCV and 3 HBV-related), 21 of hepatocellular carcinoma (both HCC and peritumoral tissues [PHCC]), and 10 controls (CONTR). Real-time PCR and Western blotting were used to measure mRNA and protein expression levels.

**Results:**

Beclin 1 mRNA levels were lower in HCC than in CH (P = 0.010) or CIRR (P = 0.011), and so were the Bcl-xL transcripts (P < 0.0001). Bad mRNA levels were higher in CH and CIRR than in CONTR, while Bax transcripts were increased in all tissues (P = 0.036). PHCC expressed the highest Bcl-2 mRNA levels. HBV-related CH tissues showed significantly higher Bcl-xL and Bad mRNA levels than HCV-related CH (P = 0.003 and P = 0.016, respectively).

**Conclusions:**

High Beclin 1, Bcl-xL and Bad levels in CH and CIRR tissues suggest an interaction between autophagy and apoptosis in the early and intermediate stages of viral hepatitis. In HCC these processes seem to be downregulated, probably enabling the survival and growth of neoplastic hepatocytes.

## Background

Autophagy (“self-digestion”) is a highly-regulated process, important in cellular homeostasis and involved in the turnover of long-lived proteins and damaged cellular organelles. Autophagy is like a two faces coin since in some cellular settings it becomes a cell survival pathway, with suppression of apoptosis, while in others, it leads to cell death, either in association with apoptosis or when apoptosis is defective [1].

Although autophagy is thought to be predominantly a cell survival mechanism, linked to a variety of physiological conditions such as aging, degenerative processes and nutrient starvation, plenty of evidence points to it having an essential role also in the pathogenesis of several human diseases. The importance of autophagy as a homeostatic and survival-promoting mechanism is indeed underscored by the demonstration of a defective autophagy in the etiology of many pathologies, such as Crohn’s disease, stroke, neurodegenerative disorders and pancreatic damage [2].

Autophagy is also involved in the carcinogenic process [3]. One of the main factors regulating autophagy is the human *beclin-1* gene, located on chromosome 17q21, monoallelically deleted in 40-75% of sporadic breast, prostate, and ovarian tumors [4]. Mice mutant for the gene coding for Beclin 1 have a relatively high incidence of spontaneous tumors, thus suggesting that a downregulated autophagy may increase the cells’ susceptibility to transformation [5]. A reduced Beclin 1 expression has been indeed demonstrated in several human cancers, including glioblastomas [6], ovarian [7], lung [8], and esophageal cancers [9]. On the other hand, Beclin 1 expression is reportedly increased in colorectal and gastric cancer cells [10,11] and these discrepant results indicate that Beclin 1 probably has different functions in different tissues.

Although the relationship between apoptosis and autophagy is still a debated topic, there are crucial proteins involved in the crosstalk involved in both processes. Many apoptotic and anti-apoptotic signals depend on interactions between Bcl-2 and other members of the Bcl-2 family. Bcl-2 is an anti-apoptotic factor that may also suppress autophagy by physically interacting with Beclin 1 [12,13], which has a so-called BH3 domain [14] that mediates the interactions with Bcl-2 and other homologs, such as Bcl-xL [15].

Beclin 1 indeed binds to Bcl-2 and Bcl-xL, through a BH3-BH3 receptor interaction and BH3-only proteins (i.e. proteins that share also with the wider Bcl-2 family the BH3 domain, that is critical for their killing capacity) stimulate autophagy by competitively disrupting this interaction and consequently leading to the release of Beclin 1 from its inhibitors.

The role of autophagy in chronic liver diseases is not well understood. To our knowledge, there are no data available on the behavior of Beclin 1 in chronic hepatitis (CH) and cirrhosis (CIRR), and very few as regards hepatocellular carcinoma (HCC), which is one of the most common and lethal tumors worldwide, and a late complication of chronic viral hepatitis and cirrhosis in more than 80% of cases [16].

The biological significance of the interaction between Beclin 1 and Bcl-2 in the liver has yet to be thoroughly explored. In this study, we evaluated Beclin 1 expression in liver tissues from patients with chronic liver diseases and HCC, looking for correlations, if any, with apoptotic and anti-apoptotic members of the Bcl-2 family.

## Methods

### Patients

#### This study involved 93 patients

A *first group* included 49 patients with chronic hepatitis (CH): 30 HCV-related 9 females, 21 males; mean age 42.6 ± 12.9 years; range 20–67; HCV-genotypes: 14 HCV-1, 8 HCV-2, 5 HCV-3, 3 HCV-4), and 19 HBV-related (3 females, 16 males; mean age 44 ± 11.1 years; range 32–63).

The *second group* included 13 cirrhotic patients (CIRR): 3 HBV-related (1 female, 2 males; mean age 52.7 ± 4.1 years; range 48–56) and 10 HCV-related (2 females, 8 males; mean age 53.1 ± 13.1 years; range 33–69).

The *third group* consisted of 21 patients with HCC in cirrhosis: 9 HCV-related (4 females, 5 males; mean age 61.2 ±15.5 years; range 23–75) and 3 HBV-related (1 female, 2 males; mean age 65.7 ±9.9 years; range 55–78); and 9 patients with HCC in non-virus-related cirrhosis (2 females, 7 males; mean age 58.8 ± 8.1 years; range 46–72).

As “control” specimens (Control), liver biopsy samples from 3 patients who had undergone cholecystectomy (2 females, 1 male; mean age 44.6±18.6 years; range 25–62) and 7 patients operated for liver metastases (4 females, 3 males; mean age 57.6±15 years; range 29–73) were considered.

The diagnosis of chronic HCV-related hepatitis was obtained on the basis of HCV-RNA positivity by polymerase chain reaction (PCR), persistently abnormal transaminases for at least 12 months, and a compatible histology. Histological diagnoses were made by a single pathologist and scored, according to Ishak’s classification [17], in all biopsy samples. Before biopsy, each patient was tested to measure HCV antibodies.

All the following studies were performed prior to any treatment.

All the patients, enrolled in this study, were recruited from the Department of Surgery, Oncology and Gastroenterology, DiSCOG, University of Padova, School of Medicine, Italy. Informed consent was obtained from each patient included in the study and the study protocol conformed to the ethical guidelines of the 1975 Declaration of Helsinki as reflected in a priori approval by the Ethical Committee of Padova University and of the Padova University Hospital Ethic Committee.

### Liver samples

During a US-guided procedure (with a 16–18 gauge modified Menghini needle), liver biopsies were obtained from patients with chronic hepatitis or liver cirrhosis according to a standard protocol. One part of the liver specimen, at least 2 cm long, was used for diagnostic purposes while the remainder was immediately frozen in liquid nitrogen and stored at −80°C.

Tissue samples were obtained from HCC at the time of surgical resection. Tumor tissues used for mRNA extraction were macroscopically selected in the middle of the nodule. The corresponding non-cancerous tissues (PHCC) were taken at least 1 cm (more where possible) from the edge of the tumor in the resected specimen. To check for any presence of infiltrating tumor cells, a slice of the sample was fixed in buffered formaldehyde, stained with haematoxylin and eosin and examined by the pathologist. In all cases, care was taken during the surgical procedure to avoid contamination between cancerous and non-cancerous samples.

All surgical liver specimens were cut into small pieces, immediately snap frozen in liquid nitrogen, then stored at −80°C.

The routine histological analysis was done in blind fashion by the pathologist. HCC lesions were classified as well-, moderately- or poorly-differentiated according to the Edmonson & Steiner criteria [18], grading the specimens on the basis of the predominant findings.

### Virological and biochemical assessments

In all HCV-infected patients, HCV-specific serum antibodies were detected by enzyme immunoassay (EIA-II; Ortho Diagnostic System) and confirmed by recombinant immunoblot assay (RIBA-II; Ortho Diagnostic System) according to the manufacturer’s instructions. HCV RNA was detected with a standardized polymerase chain reaction (PCR) (Amplicor, Roche Diagnostic Systems, Neuilly, France) in the total RNA extracted from biopsies.

HCV genotype was determined by the Inno-Lipa II HCV method (Innogenetics S.A., Gent Belgium). HCV genotypes were classified as genotype 1, subtypes 1a and 1b, and the remaining subtypes of types 2, 3, and 4 were pooled under each corresponding genotype.

HBV serum markers were tested by radio-immune assay (RIA) (Abbott, Chicago-Illinois, USA), while HBV-DNA was tested with a commercially-available fluid phase hybridization assay (Abbott, Chicago-Illinois, USA).

Data regarding transaminases were obtained.

### RNA isolation

Total RNA was extracted from frozen hepatic tissue with acid guanidium thiocyanate-phenol-chloroform according to the Chomczynski and Sacchi method [19]. RNA concentrations were quantified spectrophotometrically. Integrity of the RNA sample was assessed by electrophoresis on a 2% agarose gel (FMC Bio Product, Rockland, ME, USA) containing ethidium bromide. The quality of the RNA isolated was also assessed using the RNA 6000 Nano Assay and the Agilent 2100 bioanalyzer (Agilent Technologies, Palo Alto, CA, USA).

### Reverse transcription

For the synthesis of complementary DNA (cDNA), 2 μg of RNA were reverse transcribed in a final volume of 40 μl in the presence of 1X PCR buffer, 1 mM each of dNTPs (dATP, dTTP, dCTP, dGTP), 1U μl −1 RNase inhibitor, 2.5 μM random hexamers, and 2.5 U μl −1 of murine leukemia virus (Perkin Elmer, Foster City, CA, USA).

The reverse transcription reaction was completed at 25°C for 10 min, 42°C for 15 min and 99°C for 5 min, in a Perkin Elmer GeneAmp PCR System 2400.

The cDNA was stored at −20°C.

### Primers

Oligonucleotide primers were designed with the Primer Express software rel. 1.0 (ABI/PE Applied Biosystems, Foster City, CA, USA) and synthesized by Primm srl (San Raffaele Biomedical Science Park, Milano, Italy). Nucleotide sequences for the sense and antisense primers used for real-time PCR were: 5’-GAGGGATGGAAGGGTCTAAG-3’, 5’-GCCTGGGCTGTGGTAAGT-3’ for Beclin-1 [ENST00000361523], and the length of this amplicon was 159 bp; 5’-GGATCCAGGATAACGGAGGC-3’, 5’-CCAGATAGGCACCCAGGGT-3’ for Bcl-2 [ENST00000306316], and the length of this amplicon was 147 bp; 5’-GGCAGGCGACGAGTTTGA-3’, 5’-CCCATCCCGGAAGAGTTCAT-3’ for Bcl-xL [ENST00000376062], and the length of this amplicon was 127 bp 5’-CCTGGCACCCAGCACAA-3’, 5’-GCCGATCCACACGGAGTACT for β-actin [ENST00000158302], and the length of this amplicon was 70 bp.

5’-GGAGGATGAGTGACGAGTTTGTG-3’, 5’-GGGTGGAGTTTCGGGATGT-3’ for Bad [ENST00000265480], and the length of this amplicon was 193 bp; 5'-CTTTTGCTTCAGGGTTTCATCC-3', 5'-TTGAGACACTCGCTCAGCTTCT-3' for Bax [ENST00000356483], and the length of this amplicon was 119 bp.

### PCR product analysis

PCR products underwent vertical electrophoresis on a 0.75 mm thick, non-denaturing 6% acrylamide/bis-acrylamide gel with 5% glycerol. The silver-nitrate-stained bands were scanned on a densitometer and image analyzer system (Quantity-one- BIO-RAD Hercules, CA, USA).

### DNA purification

Purified DNAs were obtained using the MinElute PCR Purification Kit according to the manufacturer’s protocol. The concentration of the purified amplicons was quantified spectrophotometrically with the Biophotometer 6131 (Eppendorf, Hamburg, Germany) and the PicoGreen ds-DNA quantitation reagent and kits.

Fluorescence was measured with the LS-5 Luminescence Spectrometer (Perkin Elmer, Foster City, CA) using 480 nm excitation and 520 nm emission.

### Quantitative absolute real-time PCR

Real-time PCR was conducted in an ABI 7900 Sequence Detection System (Applied Biosystems, Foster City, CA, USA) using SYBR Green I [20]. The reaction was obtained on 96-well plates, in a 25μL final volume containing 1X TaqMan buffer, 5.5 mmol of MgCl_2_, 200 μmol of nucleotides, 0.01U mL-1 of AmpErase UNG, 0.25U of AmpliTaq Gold Polymerase (SYBR Green Master Mix), 300nM of each primer and 5μL of cDNA template. After one 2-min step at 50°C and a second step at 95°C for 10 min, samples underwent 45 cycles of 45 s at 94°C and then: 45 s at 60°C for Beclin-1, Bcl-X_L_, Bax and β-actin; 45 s at 62°C for Bcl-2 and 45 s at 65°C for Bad. A final extension step was performed at 60°C for 10 min. All tests were performed in triplicate. Samples in which the cDNA was omitted were used as negative controls.

### Gene expression quantification

The amounts of mRNA in the unknown samples were determined from the standard curves of the gene of interest [21]. Standards curves were generated using serial diluition (1:10) from 10^8^ to 10^2^ copies/μl of a reference sample.

Data were expressed as the ratio of the transcript amounts of the gene of interest to the β-actin transcript used as housekeeping gene.

### Western blot analysis

Total protein extracts were obtained by homogenizing liver tissues with RIPA lysis buffer (20 mM TrisHCl pH 7.4, 150 mM NaCl, 5 mM ethylene diamine tetra-acetic acid [EDTA], 1.5% Niaproof, 1 mM sodium orthovanadate Na_3_VO_4_ 0.1% sodium dodecyl sulfate [SDS]). Protein concentration was determined using the RC DC Protein Assay (Bio-Rad, Hercules, CA, USA). 40 μg of boiled proteins were loaded onto the gel. Proteins were separated by SDS-PAGE, transferred onto nitrocellulose membrane (Hybond ECL, GE Healthcare Buckinghamshire, UK) and blocked with fat-free milk [5% in Tween-phosphate-buffered saline (PBS)] for 1 h. Membranes were probed with the primary mouse monoclonal antibody against human Beclin-1 (1:200), Bcl-2 (1:1000), Bcl-X_L_ (1:200), Bad (1:500), Bax (1:500), and the primary mouse monoclonal antibody against β-actin (1:1000) (Santa Cruz Biotechnologies, California, USA). After incubation with the secondary antibody (goat anti-mouse IgG, at a dilution of 1:10000), immunoreactive proteins were visualized by chemiluminescence using SuperSignal WestPico Chemiluminescent Substrate (Pierce) and captured on X-ray film (Hyperfilm ^TM^ ECL, GE Healthcare, Buckinghamshire, UK). Molecular sizing was carried out using the Full-Range Rainbow Molecular weight Marker (GE Healthcare, Buckinghamshire, UK). Exposed films were digitized and the bands were semi-quantitatively evaluated by densitometric analysis. The Beclin-1, Bcl-2, Bcl-X_L_, Bad and Bax protein expression levels were thus normalized to those of the housekeeping gene, β-actin.

### Statistical analysis

All results are given as mean values ± SD (standard deviation). Statistical analyses were performed using the StatSoft software, rel. 5.0 (Tulsa, OK, USA). Differences between groups were analyzed with the Kruskal-Wallis test or Mann–Whitney *U* test, as appropriate. Two-tailed P value < 0.05 were deemed to be significant. The relationship between two variables was determined using Spearman’s rank correlation analysis.

## Results

**Figure 1:**
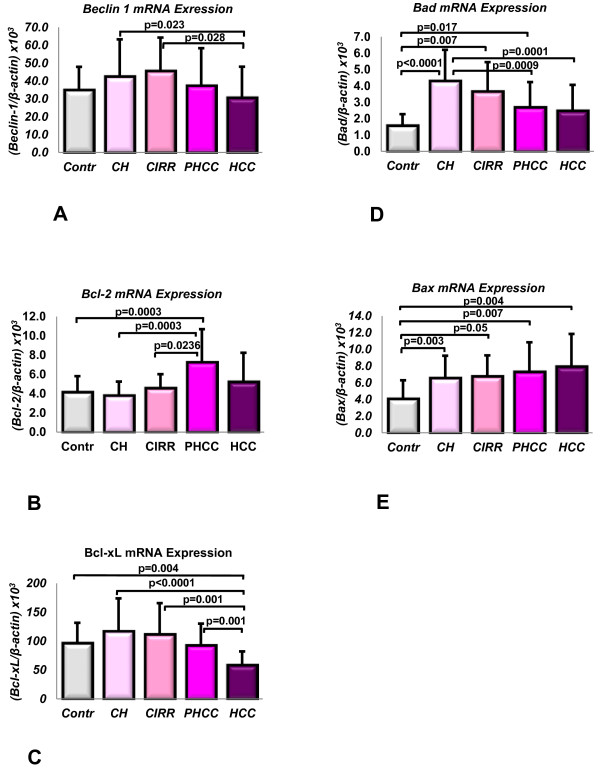
**mRNA analysis by quantitative absolute Real-Time PCR using SYBR Green I.** Gene analysis of Beclin 1 (**A**), Bcl-2 (**B**), Bcl-xL (**C**), Bad (**D**) and Bax (**E**). Control: control specimens; CH: chronic hepatitis; CIRR: cirrhosis; PHCC: cirrhotic tissues surrounding hepatocellular carcinoma; HCC: hepatocellular carcinoma. Data are expressed as mean ± SD of the ratio of the gene of interest to that of β-actin.

**Figure 2:**
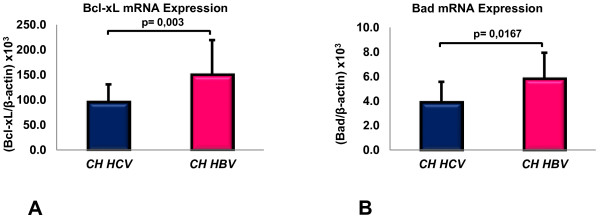
**Bcl-xL and Bad mRNA transcripts in chronic hepatitis HBV- and HCV-related tissues.** Bcl-xL **(A)** and Bad **(B)** gene expression. Data are expressed as mean ± SD.

The Beclin 1, Bcl-2, Bcl-xL, Bad and Bax mRNA levels expressed in the liver tissues are shown in Figure 1 and Figure 2.

### Beclin 1 mRNA expression

Beclin 1 mRNA levels were significantly lower in HCC tissues than in CH or CIRR (30.84 ± 17.1 vs 42.65 ± 20.6; P = 0.023 and 45.80 ± 18.4; P = 0.028, respectively), while there was no difference with PHCC tissues (Figure 1A).

There were no significant differences either in Beclin 1 mRNA levels between HCV-related versus HBV-related CH (39.67 ± 19.57 vs 47.36 ± 21.8).

### Bcl-2 mRNA expression

The Kruskal Wallis test revealed a statistically significant difference (P = 0.0003) in the Bcl-2 transcript levels among the various groups, with PHCC tissues expressing the highest Bcl-2 levels (7.28 ± 3.4) when compared with Contr (4.16 ± 1.6; P = 0.009), CH (3.83 ± 1.4; P < 0.0001) and CIRR (4.60 ± 1.3; P = 0.023) (Figure 1B).

There were no differences in Bcl-2 mRNA expression in the CH tissues in relation to the type of viral infection involved (HBV-HCV), but statistically significant differences were found between PHCC HCV (7.65 ± 3.8) and both CH HCV-infected tissues (3.79 ± 1.1; P = 0.012) and Contr (4.163.79 ± 1.1; P = 0.04).

### Bcl-xL mRNA expression

The Kruskal Wallis test showed a statistically significant difference (P < 0.0001) among the various groups. The mean Bcl-xL transcript levels were significantly lower in HCC (59.04 ± 22.9) than in PHCC (93.14 ± 37.1, P = 0.001), CIRR (112.01 ± 53.4, P = 0.001), CH (117.54 ± 56.3, P < 0.0001) or Contr (97.25 ± 34.4; P = 0.004) (Figure 1C).

A statistically significant difference was also found in the CH tissues according to the disease’s etiology, with HCV-related CH showing lower Bcl-xL expression than HBV-related CH (96.53 ± 34.2 versus 150.73 ± 68.3, P = 0.003) (Figure 2A).

These differences were confirmed also considering only HCV-infected tissues: Bcl-xL mRNA expression in HCC (64.92 ± 29.9) was significantly lower than in CIRR (107.50 ± 47.6, P = 0.05) or CH (96.53 ± 34.2, P = 0.023).

Moreover, when only cases of HCV-related hepatitis were considered, patients with HCV genotype 1 infection had a statistically lower Bcl-xL mRNA expression (75.38 ± 19.0) than cases with genotype 2 (114.57 ± 34.3, P = 0.010), or genotype 3 (104.85 ± 22.4, P = 0.019).

### Bad mRNA expression

A statistically significant difference (P < 0.0001 Kruskal Wallis test) emerged in this parameter among the five groups considered.

Contr had the lowest Bad mRNA expression (1.59 ± 0.6), and the difference was statistically significant versus CH (4.32 ± 1.8; P < 0.0001), CIRR (3.68 ± 1.7; P = 0.007) and PHCC (2.71 ± 1.51; P = 0.017). A statistically significant reduction in Bad mRNA levels was found going from CH to PHCC (P = 0.0001) and HCC (2.50 ± 1.5; P = 0.0009) (Figure 1D).

Bad mRNA levels in HBV-related CH tissues were significantly higher (5.84 ± 2.0) than in the HCV-related CH tissues (3.93 ± 1.6; P = 0.016) (Figure 2B) and patients with genotype 1 HCV (3.93 ± 1.2) expressed higher Bad mRNA levels than patients with genotype 4 (2.56 ± 0.5; P = 0.046).

### Bax mRNA expression

The Kruskal Wallis test indicated a significant difference in this parameter (P = 0.026) among the various groups. Again, Contr expressed the lowest Bax mRNA levels (4.12 ± 2.1). These levels were significantly lower than in CH (6.61 ± 2.6; P = 0.003), CIRR (6.80 ± 2.4; P = 0.05), PHCC (7.35 ± 3.4; P = 0.007) or HCC (7.99 ± 3.8; P = 0.004) (Figure 1E).

No differences emerged between HCV- and HBV-related CH cases.

Bax transcripts were significantly higher in HCV genotype 1 (7.62 ± 3.0) than in HCV genotype 3 (4.60 ±1.7; P = 0.033).

### Western blot

**Figure 3:**
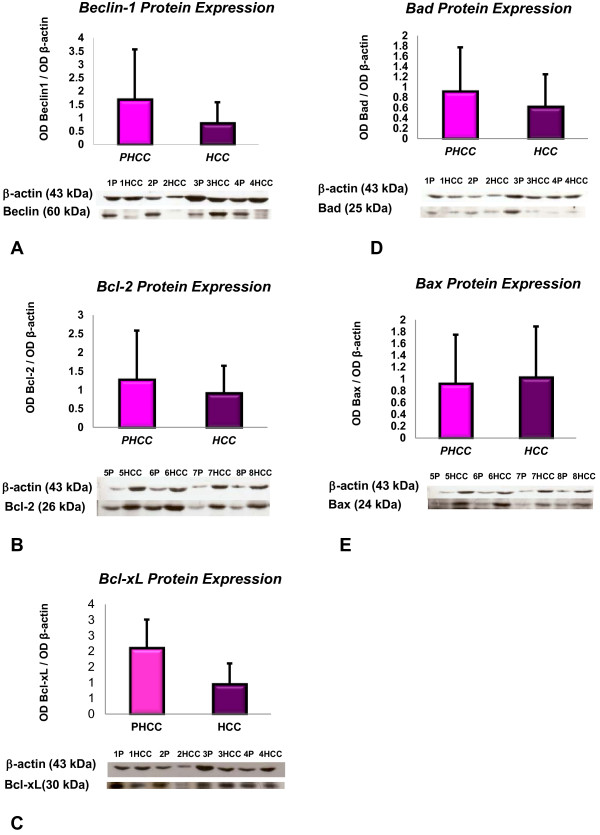
**Protein expression in HCC and the corresponding tissues surrounding HCC.** Western blot analysis of: Beclin 1 **(A)**; Bcl-2 **(B)**; Bcl-xL **(C)**; Bad **(D)**; Bax **(E)**. P: cirrhotic liver tissues surrounding hepatocellular carcinoma; HCC: tumoral tissues of 8/13 patients. Data are expressed as the optical density ratio of genes to β-actin. Values are reported as mean values ± SD.

Beclin 1, Bcl-2, Bcl-xL, Bad and Bax protein expressions were assessed only in the surgical liver tissues from 13 HCC patients (both in the cancer and in the surrounding cirrhotic tissue). Like the mRNA expression in these tissues, the Beclin 1, Bcl-2, Bcl-xL Bad and Bax protein expression levels did not differ. Low Bcl-xL protein levels were found in HCC compared to tissues surrounding tumors, however without reaching the statistical significant difference as found for Bcl-xL mRNA expression (Figure 3).

### Correlation analysis

**Figure 4:**
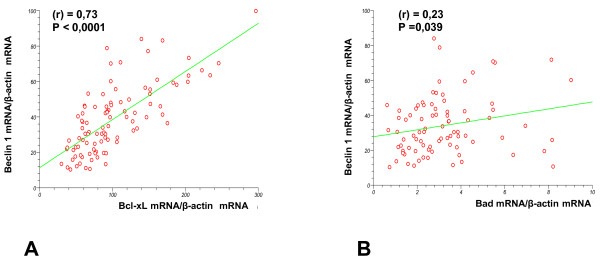
**Linear regression analysis.** Results of the linear regression analysis of: Beclin 1/β-actin vs Bcl-xL/β-actin **(A)**; Beclin 1/β-actin vs Bad/β-actin **(B)** in all tissues considered.

A significant positive correlation emerged between Beclin 1 and Bcl-xL mRNA expression in CH (r = 0.69; P < 0.0001), CIRR (r = 0.84; P = 0.0008), and HCC (r = 0.67; P = 0.002), and when all tissues were considered together (r = 0.64; P < 0.0001) (Figure 4A). This correlation was also significant when only patients with HCV infection were considered (r = 0.68; P < 0.0001). A significant positive correlation also emerged for Beclin 1 and Bcl-xL mRNA expression in HBV-related CH tissues (r = 0.88; P < 0.0001).

An inverse correlation was found between Beclin 1 and Bcl-2 in HCC tissues (r = −0.53; P = 0.03) while a positive correlation in PHCC was identified (r = 0.54; P = 0.01). Positive correlations were identified between Beclin 1 and Bax in CH (r = 0.49; P = 0.003) and also in HCV-related CH alone (r = 0.57; P = 0.002). Positive correlations were also observed between Beclin-1 and Bad when all tissues were considered (r = 0.23; P = 0.003) (Figure 4B) and in particularly in PHCC (r = 0.63; P = 0.003). While an inverse correlation was found between Bcl-2 and Bcl-xL in HCC (r = −0.58; P = 0.01).

## Discussion

In this study we evaluated the expression of Beclin 1 and of some pro- and anti-apoptotic members of the Bcl-2 family in liver tissues obtained from chronic liver disease patients in various stages in a cross-sectional study with a view to shedding light on their role in the natural history of chronic liver disease and its progression from chronic hepatitis to cirrhosis and hepatocellular carcinoma.

### Beclin 1 expression in chronic liver disease

Beclin 1 is one of the main autophagocytic agents, a regulator gene that bridges autophagy, apoptosis and differentiation. In our study, Beclin 1 mRNA expression was constitutive of all the tissues examined (in contrast with what previously described by Shi Yh et al. [22]) and the highest Beclin 1 mRNA levels were found in cirrhotic liver tissues in the absence of HCC. The significantly lower Beclin 1 mRNA expression seen in HCC by comparison with non-cancerous CH and CIRR tissues probably reflects previous observations in other types of human tumor, e.g. in ovarian, breast, prostate and lung cancer [8]. On the other hand, previous data obtained in liver studies using cDNA microarray analysis actually demonstrated an upregulated Beclin 1 mRNA expression in HCC [23]. Daniel et al. [24] compared Beclin 1 mRNA expression in HCC with adjacent non-tumor tissues and found similar mean transcript levels, although the ratio of the Beclin 1 mRNA expression in the tumor/non-tumor tissue varied considerably between different patients. Shi Yh et al. [22] found that only 31.7% of HCC had Beclin 1 expression and that HCC patients with positive Beclin 1 expression had a significantly better prognosis both in terms of disease-free and of overall survival. Using a quantitative absolute real-time PCR and Western blot analysis, respectively, we found no significant differences in Beclin 1 transcript or protein expression between HCC and surrounding tissues, while no data are available at the moment with respect to patient survival in our series. In any case, our aim was not to correlate Beclin 1 or other parameters to HCC patients survival but to describe the interactions between autophagy and apoptosis in the natural history of chronic liver disease.

The low Beclin 1 mRNA levels detected in our HCC tissues (compared with CH and CIRR without tumor) support the hypothesis that a downregulated autophagy can contribute to tumor progression. Malignant cells frequently display low levels of autophagocytic activity [25], and this may coincide with a greater chromosome instability, consequently favoring tumorigenesis [3]. On the other hand, a defective autophagy in tumor cells may result in their impaired survival in the hostile microenvironment, thus leading to tumor cell death that triggers chronic inflammation and an increased release of cytokines, which again may enhance tumor growth [25].

### Beclin 1 and HBV and HCV infection

Autophagy also has an important role in response to viral infection. HCV replication is a process occurring in association with intracellular structures, i.e. lipid droplets that interact with other organelles such as the endoplasmic reticulum (ER) [26], where HCV proteins-assembly takes place. In fact, the HCV replication is compartmentalized by lipid bilayer membranes and in infected hepatocytes, HCV replication may induce the accumulation of autophagosomes, which would interfere with autophagy itself and lead to a lysosomal fusion, inducing ER stress [27]. In our HCV-infected tissues, we found no statistically significant differences in Beclin 1 transcript levels with the progression of liver damage or the HCV genotype (as previously reported also by Ait-Goughoulte M et al. [28]), a fact that suggests that HCV viral proteins are not directly involved in modulating Beclin 1 expression. The absence of any statistically significant difference in Beclin 1 expression in tissues from patients with HCV- versus HBV-related CH suggest that the higher Beclin 1 expression levels seen in hepatitis are an epiphenomenon of inflammation, prone to a burn-out process in the more advanced phases, and that this process takes place irrespective of the type of virus involved.

### Apoptotic mediators in chronic liver diseases

To investigate the crosstalk between autophagy and apoptosis in human chronic liver disease, we analyzed pro- (Bax and Bad) and anti-apoptotic (Bcl-2, Bcl-xL) members of the Bcl-2 family.

As concerns the pro-apoptotic members, we found a downregulation of Bad levels in PHCC and HCC. BH3-only proteins, such as Bad [29], are cell death initiators The overexpression of BH3-only molecules leads to apoptosis and loss of genetic function. Bad selectively heterodimerizes with Bcl-xL and Bcl-2, but not with other family members, and when Bad binds to Bcl-xL and Bcl-2, it neutralizes their anti-apoptotic activity. Also Bax has been shown to homodimerize as well as heterodimerize with Bcl-2. Typically, a translocation of about 20% of cellular Bax from the cytoplasm to the mitochondrial outer membrane is sufficient to induce apoptosis [30]. These apoptotic and anti-apoptotic interactions contribute to the cell’s fate, and it is well known that the apoptotic pathway can be regulated by the formation of homo- and heterodimers of both apoptotic and anti-apoptotic factors [31]. Moreover "in vitro" experiments demonstrate that small differences in the amount of Bcl-2 and Bcl-xL lead to large differences in cell survival. This was particularly evident for Bcl-xL that is about ten time more efficient than Bcl-2 in preventing doxorubicin induced apoptosis in MCF-7 breast cancer cell lines [32]. The quantitative and qualitative differences in Bcl-2 and Bcl-xL protein activity in inhibiting apoptosis seems to depend by changes in their subcellular localisation.

The mitochondrial membrane represent the principal site of Bcl-2 family proteins for apoptosis regulation. However, recently Bcl-2 family members are present on the ER, were they seem to have more extensive functions. Only in the ER indeed, Bcl-2 or Bcl-xL inhibits autophagy [33].

### Interactions between Beclin 1 and apoptotic mediators in liver diseases

Beclin-1 was originally discovered as a protein interacting with Bcl-2; it contains a BH3 domain crucial to its binding to anti-apoptotic factors [34]. Beclin 1/Bcl-2 and Beclin 1/Bcl-xL heterodimers may inhibit autophagy, but only in the ER. The debate on the topic is however still open, since Priault et al. [35], using an in vitro model based on HCT116 cell lines, demonstrated that the subcellular localization of Bcl-xL was modified by starvation and, in particular, that Bcl-xL acts on the whole independently of Beclin 1, helping the formation of autophagosomes.

We did not investigate the levels of these heterodimers in our tissues, but it is clear that liver tissues with chronic liver damage (caused by either HBV or HCV infection) expressed higher levels of Beclin 1, Bcl-xL and Bad transcripts than peritumoral or tumor tissues. With respect to the type of virus involved, we observed that tissues HBV-related CH revealed higher levels of Bad and Bcl-xL mRNA transcripts than HCV-related CH, while no difference was seen in Beclin 1 expression.

The X protein of HBV (HBx) has been shown to contain a BH3 domain [36] that may interact with and inhibit Bcl-xL. Hong Tang et al. conducted in vitro studies using hepatoma cell lines and demonstrated that HBx upregulates endogenous Beclin 1 mRNA and protein expression levels [37]. In our HBV-related tissues, activation of the apoptotic pathway seems to be facilitated at the expense of autophagy, as confirmed by the predominant expression of apoptotic factors in these tissues. We need to bear in mind, however, that in HBV infection, HBx is produced in the infected cells at much lower levels than in transfection experiments.

The low levels of Beclin 1, Bcl-xL and Bad mRNA observed in HCC support their relationship in promoting tumorigenesis and favoring tumor cell progression. This relationship is confirmed by the significant positive correlations found between Beclin 1 and Bcl-xL in these tissues. A strong positive correlation between tumoral/non tumoral ratio of Beclin 1 mRNA and Bcl-xL mRNA in HCC was also reported by Daniel F et al. [24].

**Figure 5:**
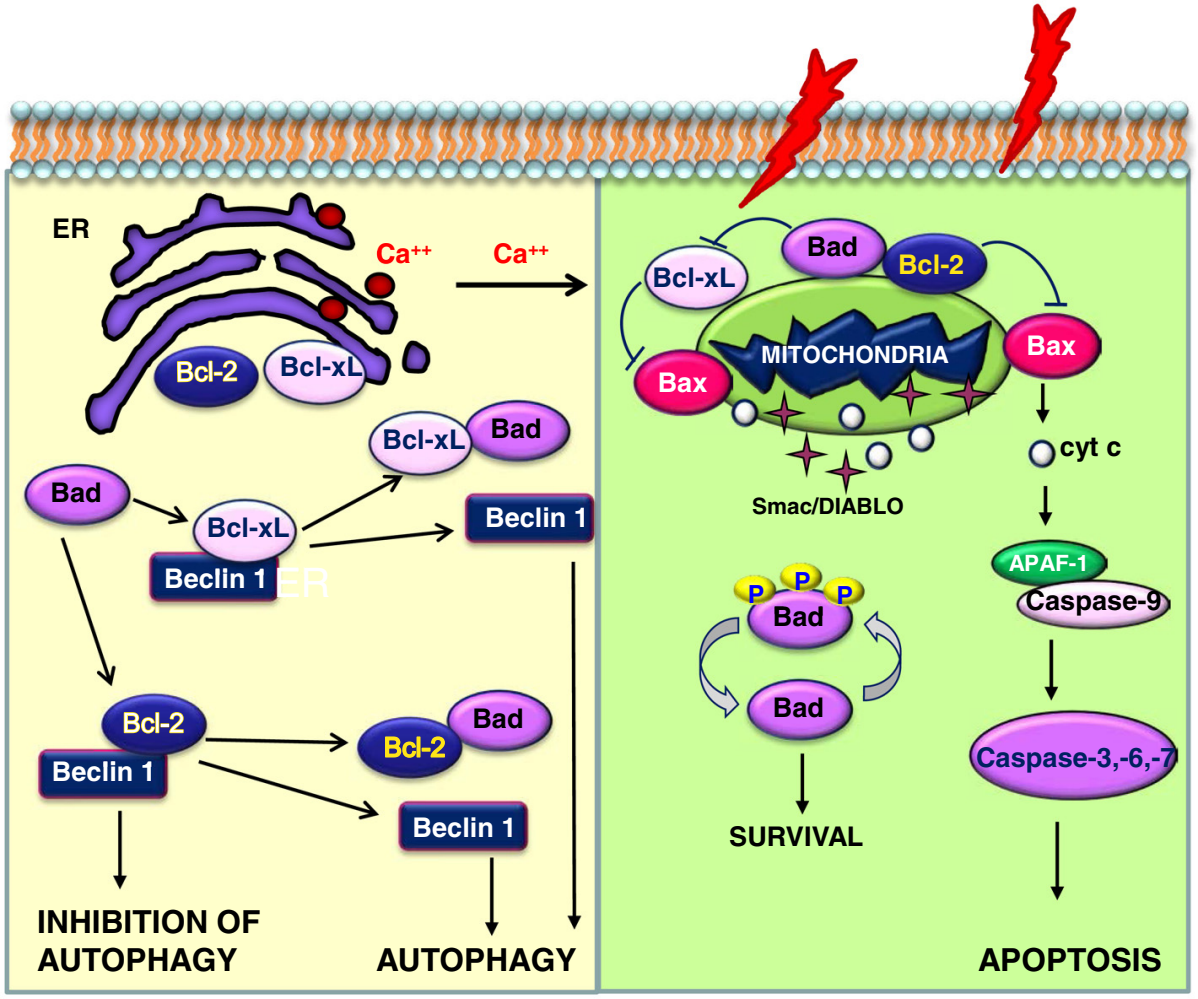
Schematic representation of the interplay between pro- and anti-apoptotic Bcl-2 family members and Beclin 1, in the endoplasmic reticulum and mitochondria.

It is worth emphasizing our observation that it was only in PHCC liver tissues that no correlations emerged between Beclin 1 and Bcl-xL, and only in these tissues that positive correlations were seen between Beclin 1 and Bad (P = 0.03) and between Bax and Bcl-2 (P = 0.01). Bax and Bak are essential for cell death, they directly induce mitochondrial dysfunction, and are often mutually supplemental, i.e. the expression of one compensates for the lack of the other [38]. On the other hand, the overexpression of Bcl-2 has been demonstrated to prevent the efflux of cytochrome c from the mitochondria and subsequent initiation of apoptosis [39] and the increase Bcl-2 expression could be important in the counteract the effect of Bax. The cross-talk between mitochondria and endoplasmic reticulum (ER) is controlled by Bcl-2 and the relative levels and interactions of Bax, Bad and Bcl-2, and their homodimerization or heterodimerization, may act like chekpoints in determining the life or death of the cell (Figure 5). This could explain the different profile of Bcl-2 and Bcl-xL expression in the respect to cirrhosis and CH. In our experience it looks like that the Bcl-2 overexpression characterizes only the late phases of the disease i.e. when HCC develops, while Bcl-xL expression begins in earlier phases of the disease itself. As seen in a previous study on the apoptotic process, it is reasonable to assume that the immunological anti-tumoral activity and cell death [40] process is concentrated mainly in the cirrhotic tissues surrounding a tumor, where the presence of large numbers of lymphocytes, monocytes/macrophages and Kupffer cells releasing large quantities of ROS and cytokines [41] may prompt the upregulation of apoptotic factors such as Bax, Bad and Bcl-2. A decrease in Beclin 1 mRNA levels would point instead to an imbalance between apoptosis and autophagy mediators probably favoring the apoptotic process. Both these processes seem to be downregulated in HCC, confirming that the survival of tumor cells in the liver relies on their evading both apoptotic and autophagocytic cell death.

## Conclusions

In conclusion, autophagy and apoptosis in the liver are interconnected processes that are overexpressed in the early and intermediate stage of viral hepatitis. The high levels of Beclin 1 observed in CH and CIRR tissues suggest a central role for autophagy as a stress-responsive pathway that may limit liver damage and interact with progression to cancer.

HBV infection seems to play a key role through the induction of expression of Bcl-xL transcripts, that are important for the neutralization of autophagic Beclin 1 protein.

The highest Bcl-2 mRNA levels and the positive correlation between Bcl-2 and Bad observed in PHCC indicate that these tissues represent a complex hepatic microenvironment were most of the inflammatory, proliferative and anti-tumoral activity takes place.

The low expression of Beclin 1, Bcl-xL and Bad we demonstrated in HCC patients points to a reduced autophagy that may favor the onset and progression of tumor.

A better understanding of the interplay between mechanisms of cell survival and cell death involved in liver disease progressing from chronic infection to cirrhosis and HCC certainly carries great potential for the development of specific treatments.

## Abbreviations

Control: Control specimens; CH: Chronic Hepatitis; CIRR: Cirrhosis; PHCC: Cirrhotic tissues surrounding Hepatocellular carcinoma; HCC: Hepatocellular carcinoma; HBV: Hepatitis B Virus; HCV: Hepatitis C Virus; ER: Endoplasmic Reticulum.

## Competing interests

The authors declare that they have no competing interests.

## Authors’ contributions

KA performed the majority of experiments; FF involved in critical reading and helpful discussion of the manuscript; CR provided the collection of human liver biopsies; CU and ND provided the collection of human liver tissues; BM designed the study and wrote the manuscript. All authors read and approved the final manuscript.

## Pre-publication history

The pre-publication history for this paper can be accessed here:

http://www.biomedcentral.com/1471-230X/12/118/prepub
